# 2-benzothiazoleacetonitrile based two-photon fluorescent probe for hydrazine and its bio-imaging and environmental applications

**DOI:** 10.1038/s41598-017-01656-w

**Published:** 2017-05-08

**Authors:** Jian-Yong Wang, Zhan-Rong Liu, Mingguang Ren, Weiying Lin

**Affiliations:** Institute of Fluorescent Probes for Biological Imaging, School of Chemistry and Chemical Engineering, School of Materials Science and Engineering, University of Jinan, Jinan, Shandong 250022 P.R. China

## Abstract

A novel turn-on two-photon fluorescent probe **NS-N**_**2**_ **H**_**4**_ was developed with the 2-benzothiazoleacetonitrile as a new recognition site for the detection of hydrazine (N_2_H_4_). The two-photon probe exhibited favorable properties including high selectivity, low cytotoxicity and almost 16-fold fluorescence enhancement in the presence of N_2_H_4_ in solution. The probe could be used to image hydrazine in the living cells. Notably, we also used the two-photon fluorescent probe to image hydrazine in the tissue imaging for the first time. Furthermore, by the way of probe-loaded TLC plate, we further monitored vapor of hydrazine. Therefore, the novel two-photon probe is expected to be employed to detect N_2_H_4_ in biosamples and environmental pollution and the new recognition site will be widely applied to construct fluorescent probes for the detection of N_2_H_4_.

## Introduction

Hydrazine (N_2_H_4_) has been widely employed in space system as rocket propellant due to its special chemical properties including flammability and explosion^1^. According to its basic and reductive properties, hydrazine has been used as catalyst, corrosion inhibitor, and reducing agent in pharmaceutical, agricultural, and applied chemical industries^2–4^. However, it is also regarded as an important industrial pollutant to humans and animals with high toxicity, which could cause the lungs, livers, and kidneys cancerous^5^. Thus, the concentration of N_2_H_4_ must be controlled as low as 10 ppb^6^. Therefore, it is highly significant to develop powerful means for the tracking and detection of N_2_H_4_ in living systems with high sensitivity and good selectivity.

There are some analytical methods for the detection of N_2_H_4_, which were exploited in the previous work, such as including chromatography-mass spectrometry, titrimetry and electro-chemical methods^7, 8^. However, sophisticated instrumentation and highly personal operating techniques must be needed in these processes, which are complex and time-consuming. In the past few decades, organic fluorescent probes, which were regarded as the most powerful monitoring tools, have become an important tool used in biological studies with excellent merits including high sensitivity, good selectivity and real-time detection^9–12^.

Very recently, a number of fluorescent probes for monitoring N_2_H_4_ in living biosystem have been reported^13–34^, most of which were reported by deprotection of the leaving group for the detection of hydrazine^13–27^. Also, only few examples were developed by the cleavage of carbon-carbon double bond^28–30^. Besides, some fluorescent probes were used for the detection of N_2_H_4_ by the way of open ring, closed ring and effect of ESIPT after reacting with N_2_H_4_^31–34^. Hence, it is very crucial to develop a new recognition site for the detection of N_2_H_4_. Furthermore, all the previous probes were excited by one-photon wavelengths leading to photobleaching of fluorescent dyes and damage to living cells and tissues. Although the two-photon confocal microscopes are relatively not common, there are significant merits of two-photon microscopy (TPM) with long excitation wavelengths such as three-dimensional detection of living tissues, depressed the photodamage to biological samples, increased penetration ability of tissue and reduced fluorescent interference of background. Therefore, it is very important and necessary to construct two-photon fluorescent probe, which could be suitable for imaging N_2_H_4_ specifically in living cells and tissues.

**Figure 1:**
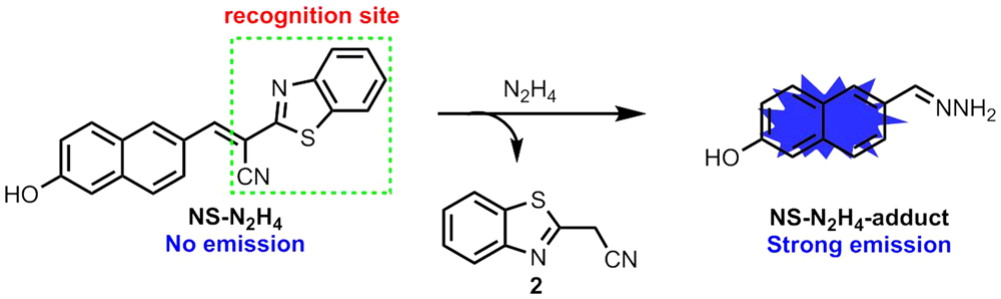
The structure of **NS-N**_**2**_ **H**_**4**_ and the proposed sensoring mechanism for N_2_H_4_.

In this report, we have constructed a novel two-photon fluorescent probe **NS-N**_**2**_ **H**_**4**_ for the detection of N_2_H_4_ with 2-benzothiazoleacetonitrile as a new recognition site. (Fig. 1) The novel turn-on fluorescent probe **NS-N**_**2**_ **H**_**4**_ was designed for the recognition of N_2_H_4_ with good selectivity over other analytes. Besides, the cell imaging and the first tissue imaging confirmed that the probe **NS-N**_**2**_ **H**_**4**_ can be used to monitor the level of N_2_H_4_ in living biosystem. Furthermore, the probe **NS-N**_**2**_ **H**_**4**_ could monitor vapor of hydrazine by the way of probe-loaded TLC plate. Therefore, the two-photon probe is expected to be employed to detect N_2_H_4_ in biosamples and environmental pollution.

## Results and Discussion

### Design and synthesis

**Figure 2:**
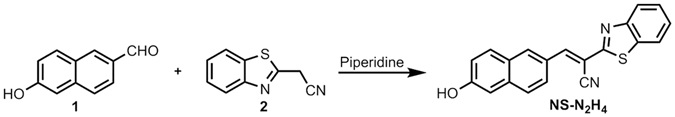
Synthesis of the two-photon fluorescent probe **NS-N**_**2**_ **H**_**4**_.

The platform of 6-hydroxy-2-naphthaldehyde was chosen as the fluorescence reporting group due to two-photon properties and easy modification. By engineering a new recognition moiety of 2-benzothiazoleacetonitrile to the fluorescent platform, we designed and synthesized the two-photon probe **NS-N**_**2**_ **H**_**4**_, which was outlined in Fig. 2 by condensation reaction in one step easily with good yield. The structure of target compound **NS-N**_**2**_ **H**_**4**_ was fully characterized by ^1^H NMR, ^13^C NMR and HRMS.

### Fluorescent properties of NS-N_2_H_4_

**Figure 3:**
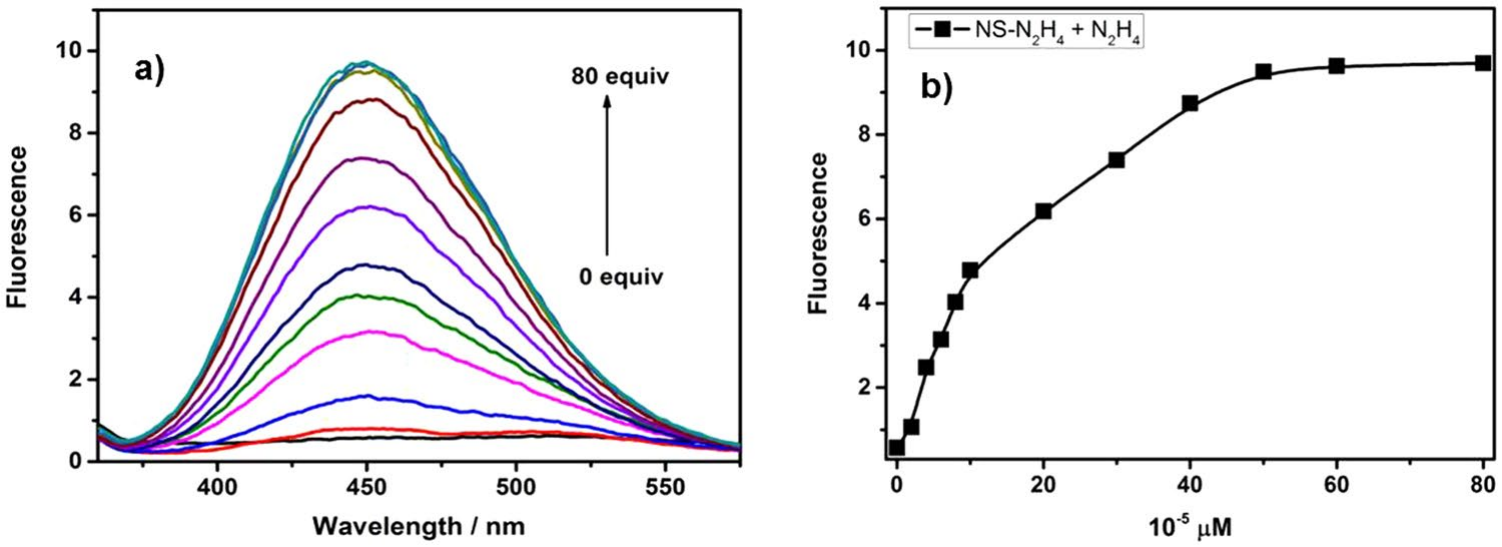
Fluorescence spectra of **NS-N**_**2**_ **H**_**4**_ (10 μM) in pH 7.4 PBS/DMSO (v/v = 2/1) in the absence or presence of N_2_H_4_.

With the two-photon probe **NS-N**_**2**_ **H**_**4**_ in hand, its optical properties were measured in the absence or presence of N_2_H_4_ including absorption (Fig. S1) and fluorescence spectroscopy. The probe **NS-N**_**2**_ **H**_**4**_ showed almost no fluorescence with excitation at 360 nm (Fig. 3). In presence of N_2_H_4_, the probe **NS-N**_**2**_ **H**_**4**_ exhibited strong emission at 448 nm in PBS-DMSO (v/v = 2/1, pH = 7.4) at ambient temperature. That is to say, PBS-DMSO (v/v = 2/1, pH = 7.4) was regarded as the suitable solvent for the fluorescence experiment. With the time extended, the fluorescence intensity was increased gradually (Fig. 3). Notably, a large fluorescence enhancement (up to 16-fold) was shown. The two-photon probe showed relatively high sensitivity in presence of N_2_H_4_ under the experimental conditions, indicating that the probe could be used as a practical tool for the detection of N_2_H_4_. In addition, the probe **NS-N**_**2**_ **H**_**4**_ is also stable under irradiation depicted in the Fig. S2.

### Mechanism

To get insight into the proposed sensing process, we studied the reaction of **NS-N**_**2**_ **H**_**4**_ with N_2_H_4_ by mass spectrometry. When the probe **NS-N**_**2**_ **H**_**4**_ (20 μM) was treated with N_2_H_4_ (400 μM) in pH 7.4 PBS/DMSO (v/v = 2/1) at room temperature, a significant peak at m/z 187.0875 corresponding to the product **NS-N**_**2**_ **H**_**4**_-adduct appears in the ESI-MS spectrum (Fig. S4), in good agreement with the proposed sensing mechanism (Fig. 1).

### Effect of pH

**Figure 4:**
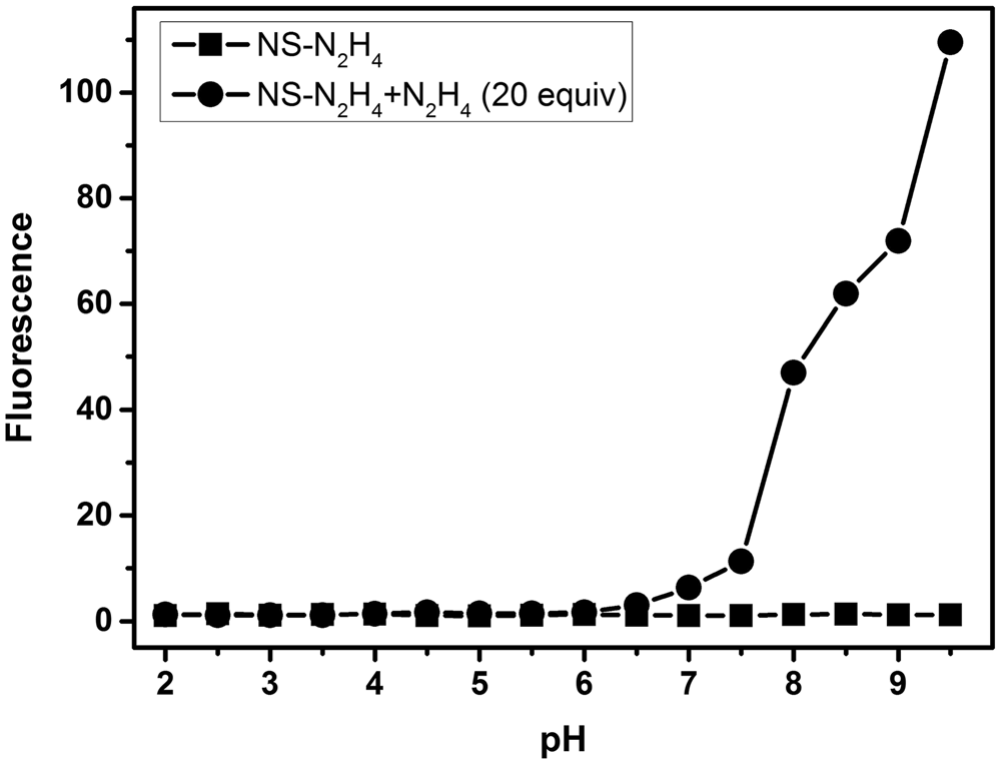
The emission intensity changes of **NS-N**_2_ **H**_4_ (10 μM) upon addition of N_2_H_4_ (20 equiv) at different pH PBS buffer, containing 33.3**%** DMSO as a cosolvent.

We then decided to examine the effect of pH on the fluorescence response of **NS-N**_**2**_ **H**_**4**_ to N_2_H_4_. As shown in Fig. 4, the emissions intensities of **NS-N**_**2**_ **H**_**4**_ are quite low and do not change significantly over wide ranges of pH from 2.0–9.5, indicating that the free probe was stable in the wide pH range. Upon treated **NS-N**_**2**_ **H**_**4**_ with N_2_H_4_, we found that the pH value of solution has a great influence on the probe **NS-N**_**2**_ **H**_**4**_ response to N_2_H_4_. With the increase of pH from 7.0 to 9.5, an enhancement trend is observed in **NS-N**_**2**_ **H**_**4**_ fluorescence intensity of response to N_2_H_4_, which covers well the physiological pH range of mitochondria (about pH 7.99), indicating that the free probe is suitable for detecting N_2_H_4_ in living cells and tissues.

### Response rate and selectivity

**Figure 5:**
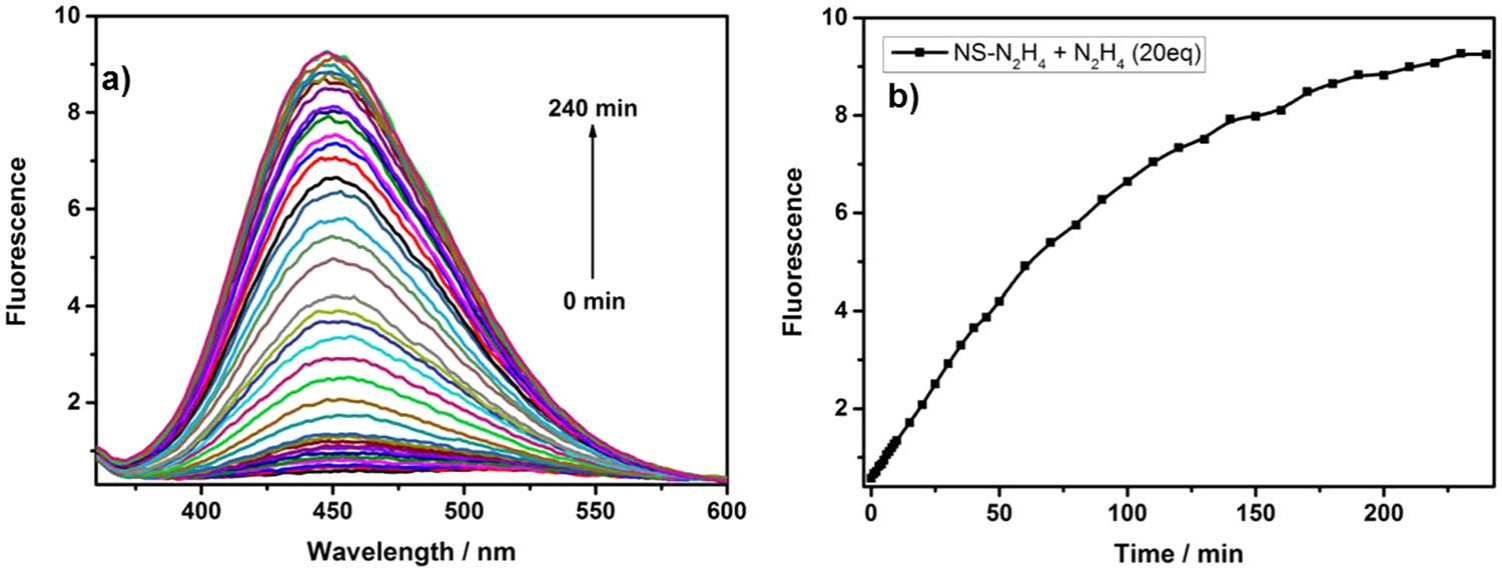
Fluorescence spectra of **NS-N**_2_ H _4_ (10 μM) in pH 7.4 PBS/DMSO (v/v = 2/1) in the absence or presence of N_2_H_4_ (20 equiv).

**Figure 6:**
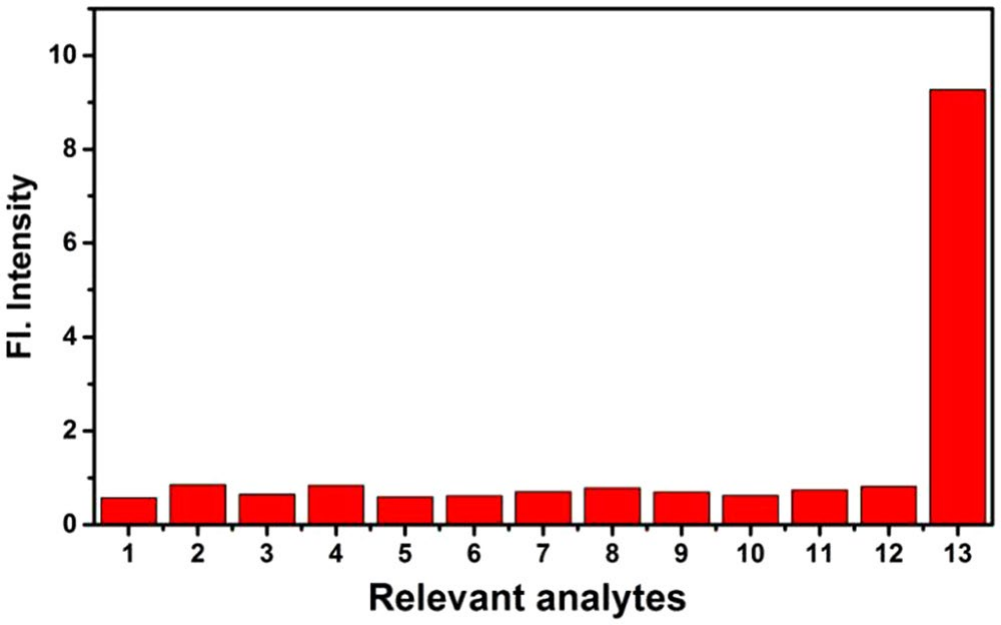
The fluorescence intensity of probe **NS-N**_**2**_ **H**_**4**_ (10 μM) in the presence of various analytes (**1**0 equiv) in PBS buffer (pH 7.4 PBS/DMSO (v/v = 2/1)). 1: none; 2: Al^3+^; 3: Ca^2+^; 4: Co^2+^; 5: Cu^+^; 6: Cu^2+^; 7: Mg^2+^; 8: Zn^2+^; 9: S^2−^; 10: SO_3_^2−^; 11: Cys; 12: Cl^−^; 13: N_2_H_4_.

The time courses of the fluorescence intensities of **NS-N**_**2**_ **H**_**4**_ (10 µM) in the presence of N_2_H_4_ (20 equiv) in pH 7.4 PBS/DMSO (v/v = 2/1) was measured in Fig. 5. Notably, a gradual increase in fluorescence intensity was observed in the presence of N_2_H_4_ in 240 min at ambient temperature. However, the fluorescence intensity increased rapidly at 37 °C (Fig. S3). The fact is that the probe **NS-N**_**2**_ **H**_**4**_ could be fit for the detection of N_2_H_4_ in real time. The high selectivity to the target molecule over other potentially competing molecules is another important property for a bioimaging probe with potential application in the biosystem. Therefore, we had performed some research on the selectivity of the free probe **NS-N**_**2**_ **H**_**4**_ with various relevant analytes including anions, metal ions, reducing agents, small molecule thiols, and N_2_H_4_ to investigate the selectivity. As shown in Fig. 6, When other analytes such as Al^3+^, Ca^2+^, Co^2+^, Cu^+^, Cu^2+^, Mg^2+^, Zn^2+^, S^2−^, SO_3_^2−^, Cys, Cl^−^ were treated with **NS-N**_**2**_ **H**_**4**_, the fluorescence intensity was almost unchanged compared with a strong fluorescent response when treated with N_2_H_4_. These results suggest that the probe **NS-N**_**2**_ **H**_**4**_ is highly selective for N_2_H_4_ over other tested species.

### Application in vapor gas detection

**Figure 7:**
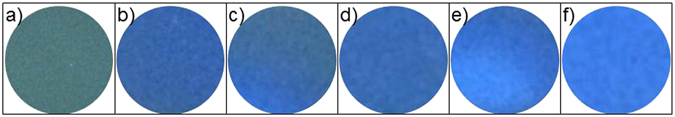
Photographs of TLC plates, soaked in the solution of **NS-N**_**2**_ **H**_**4**_ followed by addition of different amounts of hydrazine. (**a**): water; (**b**): 10% N_2_H_4_; (**c**) 30% N_2_H_4_; (**d**) 40% N_2_H_4_; (**e**) 50% N_2_H_4_; (**f**) 80% N_2_H_4_.

Encouraged by the above excellent properties of the probe **NS-N**_**2**_ **H**_**4**_, we evaluated its potential utility for the detection of hydrazine in real samples. At the beginning, TLC plates were soaked in the solution of **NS-N**_**2**_ **H**_**4**_ (0.1 mM, in DMSO). After dried, the **NS-N**_**2**_ **H**_**4**_ probe-loaded TLC plates were used to detect gaseous hydrazine, which can further discriminate hydrazine aqueous solutions with different concentrations (Fig. 7). When exposed to the vapor of hydrazine for 10 min, distinctive fluorescence color changes of **NS-N**_**2**_ **H**_**4**_-loaded TLC plates were observed (Fig. 7b–f), which were highly dependent on the concentration of hydrazine in aqueous solution and easy to be distinguished by the naked eyes. However, no visible change was observed by applying blank solvent (distilled H_2_O, Fig. 7a). Therefore, these results demonstrate that the probe **NS-N**_**2**_ **H**_**4**_ is suitable for the instant visualization of trace amounts of hydrazine in environmental samples

### Bioimaging in living cells

The above measurements indicate that the two-photon fluorescent probe has good properties including sensing appropriately at physiological pH, a very large turn-on signal, in particular a new recognition site, good selectivity. Thus, the probe **NS-N**_**2**_ **H**_**4**_ seems to be fit for the detection of N_2_H_4_ in real biosamples. We evaluated **NS-N**_**2**_ **H**_**4**_ imaging assays in live cells, and fluorescence imaging experiments were carried out in living cells (HeLa cells) on confocal laser scanning microscopy.

The cytotoxicity of **NS-N**_**2**_ **H**_**4**_ was examined toward Hela cells by a MTT assay (see Supplementary Fig. S5). The results have proved to be that the probe **NS-N**_**2**_ **H**_**4**_ at the low concentrations has no marked cytotoxicity to the cells after a long period (>90% HeLa cells survived after 24 h with **NS-N**_**2**_ **H**_**4**_ (30.0 µM) incubation). Therefore, the probe **NS-N**_**2**_ **H**_**4**_ is suitable for imaging N_2_H_4_ in living cells due to the low cytotoxicity.

**Figure 8:**
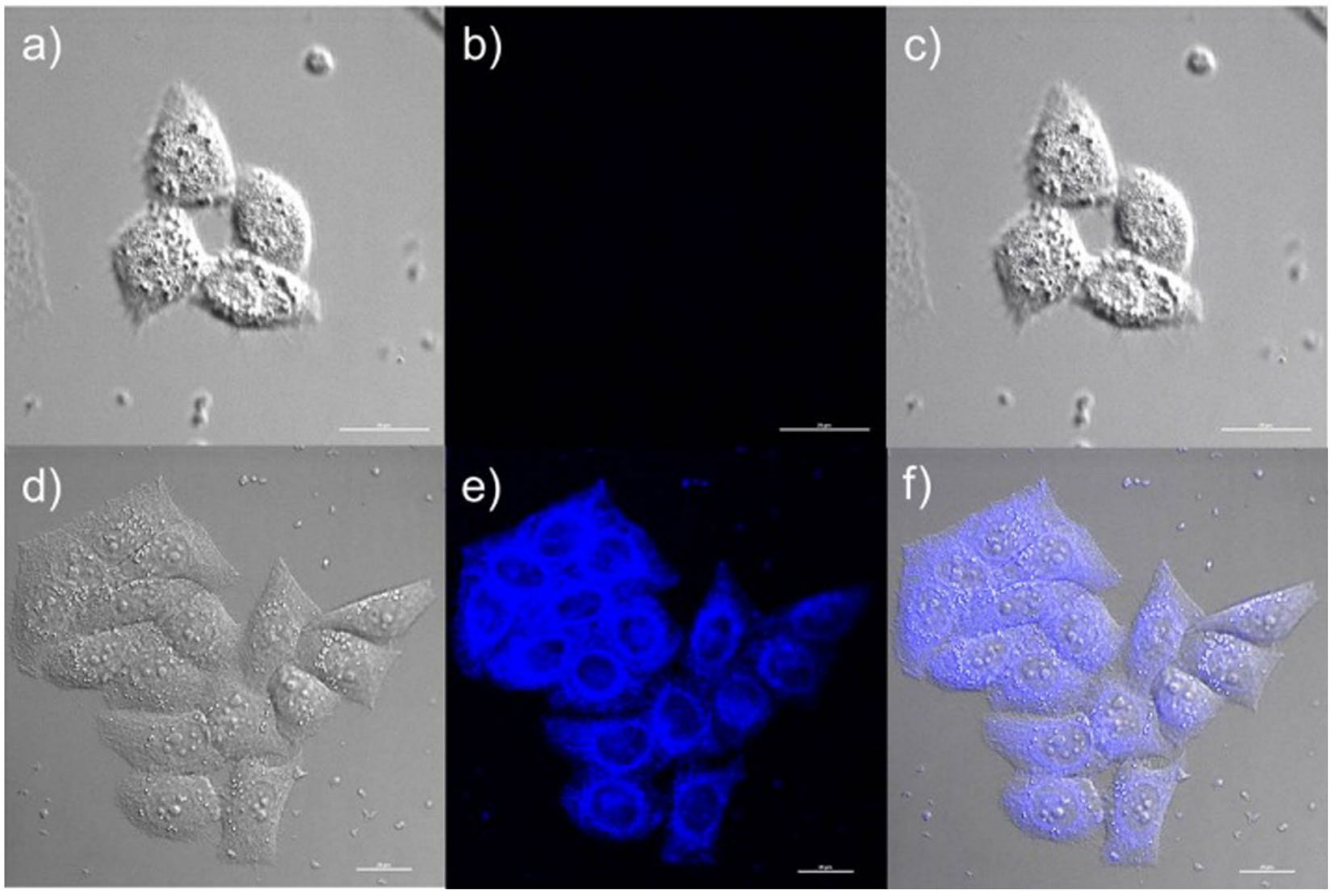
Brightfield and fluorescence images of HeLa cells stained with the probe **NS-N**_**2**_ **H**_**4**_. (**a**) Brightfield image of HeLa cells costained only with **NS-N**_**2**_ **H**_**4**_; (**b**) Fluorescence images of (**a**) from blue channel; (**c**) overlay of (**a** and **b**); (**d**) Brightfield image of HeLa cells costained with **NS-N**_**2**_ **H**_**4**_ and treated with N_2_H_4_; (**e**) Fluorescence images of (**d**) from blue channel; (**f**) overlay of the brightfield image (**d**) and blue channels (**e**).

After established the excellent sensing performance and the low cytotoxicity of the probe **NS-N**_**2**_ **H**_**4**_, we examine whether the probe could be functional in living cells. The utility of **NS-N**_**2**_ **H**_**4**_ for fluorescence imaging of N_2_H_4_ in living cells was investigated (Fig. 8). When HeLa cells were incubated with **NS-N**_**2**_ **H**_**4**_ for 30 min, no detectable fluorescence was observed. However, when the cells were pre-treated with **NS-N**_**2**_ **H**_**4**_ for 30 min and then incubated with N_2_H_4_ (10 equiv) solution for another 30 min, the strong fluorescence was shown in the blue channel (Fig. 8e) at the same test conditions, confirming that the probe possess good membrane permeability and could image N_2_H_4_ in cellular environment.

### Bioimaging in living tissues

**Figure 9:**
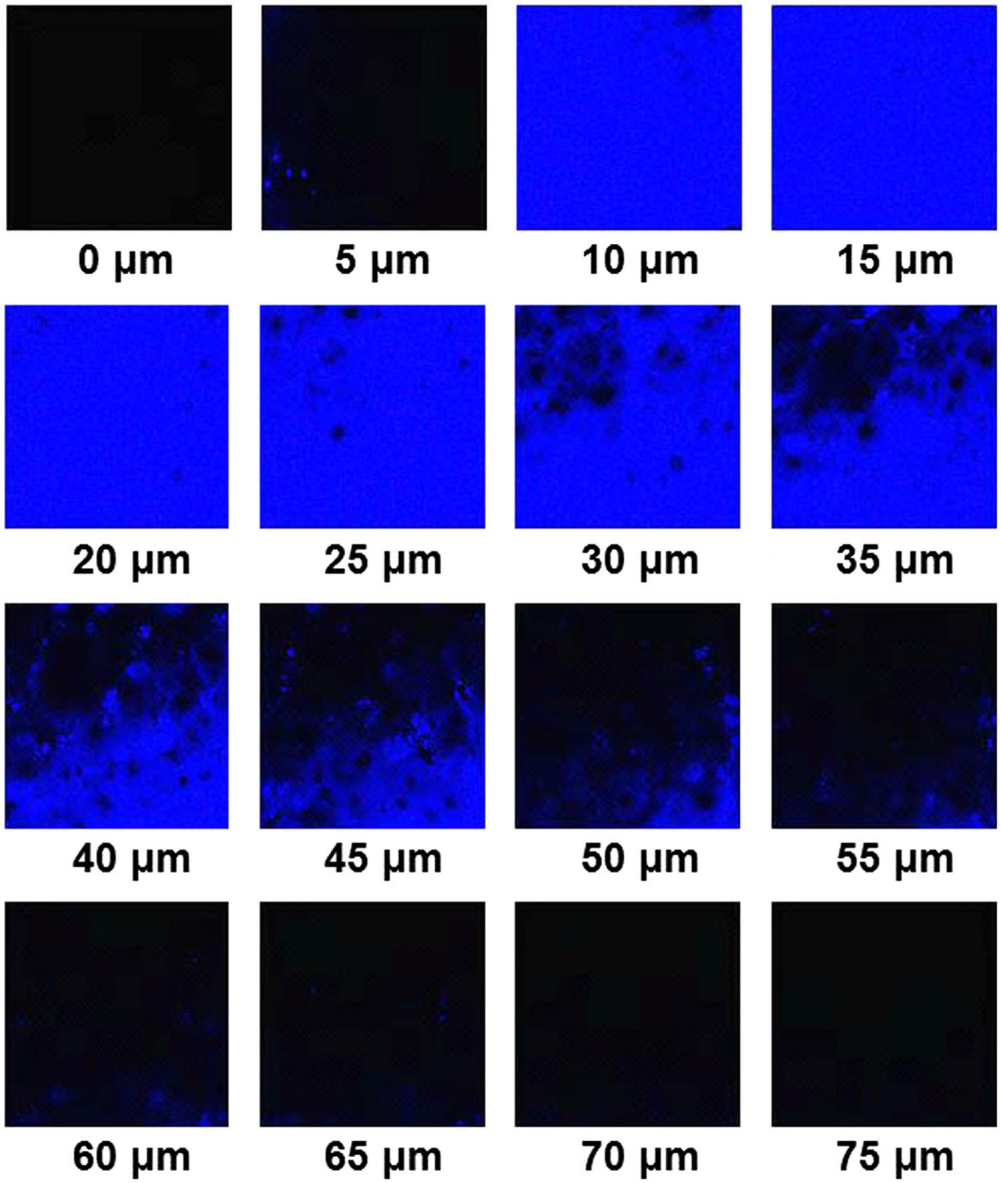
Two-photon fluorescence images of a fresh mouse liver slice pretreated with **NS-N**_**2**_ **H**_**4**_ (20 μM) and then with N_2_H_4_ (20 equiv) in PBS buffer at the depths of approximately 0–75 μm. Excitation at 800 nm with fs pulse.

Encouraged by the above ideal results of the probe in the blue channel for monitoring N_2_H_4_ and the advantages of two-photon fluorescence microscopy, we decided to further operate experiment for the detection of N_2_H_4_ in living tissues by two-photon fluorescence microscopy. The living tissues slices of the fresh rat liver were prepared with thickness at 400 μm, which were measured by two-photon fluorescence microscopy. At the beginning, tissue slices incubated with only the probe **NS-N**_**2**_ **H**_**4**_ (20.0 μM) for 30 min at 37 °C in PBS exhibit no fluorescence at the emission window of 0–75 nm (Fig. S6). When tissue slices were incubated with **NS-N**_**2**_ **H**_**4**_ (20.0 μM) for 30 min, and then treated with N_2_H_4_ (20 equiv) for another 30 min, significant fluorescence emerged up to 75 μm depth of living tissues by the way of two-photon fluorescence microscopy, which has exhibited its two-photon fluorescence properties (Fig. 9).

## Conclusions

In conclusion, we have developed a turn-on two-photon fluorescent probe with the 2-benzothiazoleacetonitrile as a new recognition site for the detection of hydrazine N_2_H_4_. Desirable properties including good selectivity and low cytotoxicity are emerged. The probe **NS-N**_**2**_ **H**_**4**_ could be used to image hydrazine in living cells as well as in living tissues for the first time. At last, the novel probe was applied to monitor vapor of hydrazine by the way of probe-loaded TLC plate. We expect that the novel probe **NS-N**_**2**_ **H**_**4**_ could be helpful for investigation and detection of N_2_H_4_ in living organisms and environmental pollution and many other fluorescent probes would be developed with this new recognition site in the future.

## Methods

### Fluorometric analysis

Without other noted, all the tests were operated according to the following procedure. A stock solution (1.0 mM) of **NS-N**_**2**_ **H**_**4**_ was prepared in DMSO. In a 10 mL tube the test solution of compounds **NS-N**_**2**_ **H**_**4**_ was prepared by placing 0.09 mL of stock solution, 3 mL of DMSO, 6 mL of 0.1 M PBS buffer and an appropriate volume of **N**_**2**_ **H**_**4**_ sample solution. After adjusting the final volume to 10 mL with 0.1 M PBS buffer, standing at room temperature 3 min, 3 mL portion of it was transferred to a 1 cm quartz cell to measure absorbance or fluorescence. All fluorescence measurements were conducted at room temperature on a Hitachi F4600 Fluorescence Spectrophotometer. The slight pH variations of the solutions were achieved by adding the minimum volumes of NaOH (0.1 M) or HCl (0.2 M).

### Vapor gas detection

TLC plates were soaked in the solution of **NS-N**_**2**_ **H**_**4**_ (0.1 mM, in DMSO). After dried, the **NS-N**_**2**_ **H**_**4**_ probe-loaded TLC plates were placed to cover a flask containing different concentration of N_2_H_4_ for 10 min at room temperature before observation.

### Cytotoxicity assay

The living cells line were treated in DMEM (Dulbecco’s Modified Eagle Medium) supplied with fetal bovine serum (10%, FBS), penicillin (100 U/mL) and streptomycin (100 μg/mL) under the atmosphere of CO_2_ (5%) and air (95%) at 37 °C. The HeLa cells were then seeded into 96-well plates, and 0, 1, 5, 10, 20, 30 μM (final concentration) of the probe **NS-N**_**2**_ **H**_**4**_ (99.9% DMEM and 0.1% DMSO) were added respectively. Subsequently, the cells were cultured at 37 °C in an atmosphere of CO_2_ (5%) and air (95%) for 24 hours. Then the HeLa cells were washed with PBS buffer, and DMEM medium (100 μL) was added. Next, MTT (10 μL, 5 mg/mL) was injected to every well and incubated for 4 h. Violet formazan was treated with sodium dodecyl sulfate solution (100 μL) in the H_2_O-DMF mixture. Absorbance of the solution was measured at 570 nm by the way of a microplate reader. The cell viability was determined by assuming 100% cell viability for cells without **NS-N**_**2**_ **H**_**4**_.

### HeLa Cells culture

HeLa cells were grown in modified Eagle’s medium (MEM) replenished with 10% FBS with the atmosphere of 5% CO_2_ and 95% air at 37 °C for 24 h. The HeLa cells were washed with PBS when used. HeLa cells treated with **NS-N**_**2**_ **H**_**4**_ (20.0 μM) for 30 min, then with N_2_H_4_ (200.0 μM) for 30 min at 37 °C. The ideal fluorescence images were acquired with a Nikon A1MP confocal microscopy with the equipment of a CCD camera.

### Tissue imaging

The Kunming mice were purchased from Shandong University Laboratory Animal Center (Jinan, China). All procedures for this study were approved by the Animal Ethical Experimentation Committee of Shandong University according to the requirements of the National Act on the use of experimental animals (China).The fresh mouse liver slices were obtained from the liver of 14-day-old mouse. The living liver slices were gained with 400 micron thickness using a vibrating-blade microtome in 25 mM PBS (pH 7.4). The living liver slices were pre-treated with **NS-N**_**2**_ **H**_**4**_ (20 μM) for 30 min. The slices were washed three times by PBS buffer and imaged. To obtain the two-photon fluorescence images of the tissues incubated with both the probe and anlysis sample (N_2_H_4_), the slices were pre-treated with **NS-N**_**2**_ **H**_**4**_ (20 μM) for 30 min before the N_2_H_4_ was added. Following this incubation for another 30 min at 37 °C, the slices were washed three times by PBS buffer and imaged. The two-photon fluorescence emission was collected at between 420 and 495 nm upon excitation at 800 nm with a femtosecond laser.

### Synthesis of the probe NS-N_2_H_4_

A mixture of 6-hydroxy-2-naphthaldehyde (0.5 mmol, 100.0 mg, 1.0 equiv) and benzothiazole-2-acetonitrile (0.55 mmol, 58.9 mg, 1.1 equiv) were dissolved in EtOH (5.0 mL). The piperidine (0.55 mmol, 46.8 mg, 1.1 equiv) was added under N_2_. After stirred at room temperature for 8 h, the reaction mixture was adjusted to distilled water (2.0 mL), and then extracted with ethyl acetate. The organic layer was washed with saturated sodium chloride, dried over Na_2_SO_4_, filtered, and concentrated under vacuum, and the product was obtained by silica column chromatography to give the probe **NS-N**_**2**_ **H**_**4**_ in the yield of 83%. ^1^H NMR (400 MHz, DMSO-*d*_6_) δ 10.4 (s, 1H), 8.49 (d, *J* = 12.4 Hz, 2H), 8.22–8.19 (m, 2H), 8.09 (d, *J* = 8.0 Hz, 1H), 7.90 (dd, *J* = 14.4, 7.6 Hz, 2H), 7.60 (t, *J* = 8.0 Hz, 1H), 7.52 (t, *J* = 7.2 Hz, 1H), 7.23–7.16 (m, 2H). ^13^C NMR (101 MHz, DMSO-*d*_6_) δ 164.6, 159.3, 153.9, 149.3, 137.6, 135.2, 134.7, 132.2, 128.1, 128.0, 127.9, 127.7, 125.8, 123.9, 123.4, 120.9, 117.6, 110.2, 104.0; HRMS (ESI) m/z calcd for C_20_H_13_ON_2_ S^+^ (M + H)^+^: 329.0743; found 329.0744.

## Electronic supplementary material

Supporting Information
