# Supplementary material for: 2-benzothiazoleacetonitrile based two-photon fluorescent probe for hydrazine and its bio-imaging and environmental applications
Source: Sci Rep. 2017 May 8;7:1530. doi: 10.1038/s41598-017-01656-w (PMC5431551; doi:10.1038/s41598-017-01656-w)
Supplement: Supplementary file 1 — Supporting Information [file 41598_2017_1656_MOESM1_ESM.doc]

*Supporting Information for*

2-benzothiazoleacetonitrile based two-photon fluorescent probe for hydrazine and its bio-imaging and environmental applications

Jian-Yong Wang, Zhan-Rong Liu, Mingguang Ren, Weiying Lin*

Institute of Fluorescent Probes for Biological Imaging, School of Chemistry and Chemical Engineering, School of Materials Science and Engineering, University of Jinan, Jinan, Shandong 250022, P.R. Email: [weiyinglin2013@163.com](mailto:weiyinglin2013@163.com)

*Correspondence to: Weiying Lin, Institute of Fluorescent Probes for Biological Imaging, School of Chemistry and Chemical Engineering, School of Materials Science and Engineering, University of Jinan, Jinan, Shandong 250022, P.R. China. Email: weiyinglin2013@163.com.

**Table of contents**

Figure S1…………………………… …………………………………………….S3

Figure S2…………………………… …………………………………………….S3

Figure S3………………………………………………………………….……….S4

Figure S4………………………………………………………………….……….S4

Figure S5…………………………………………………………………………..S5

Figure S6…………………………………………………………………………..S5

Figure S7…………………………………………………………………………..S6

Figure S8…………………………………………………………………………..S6

Figure S9…………………………………………………………………………..S7


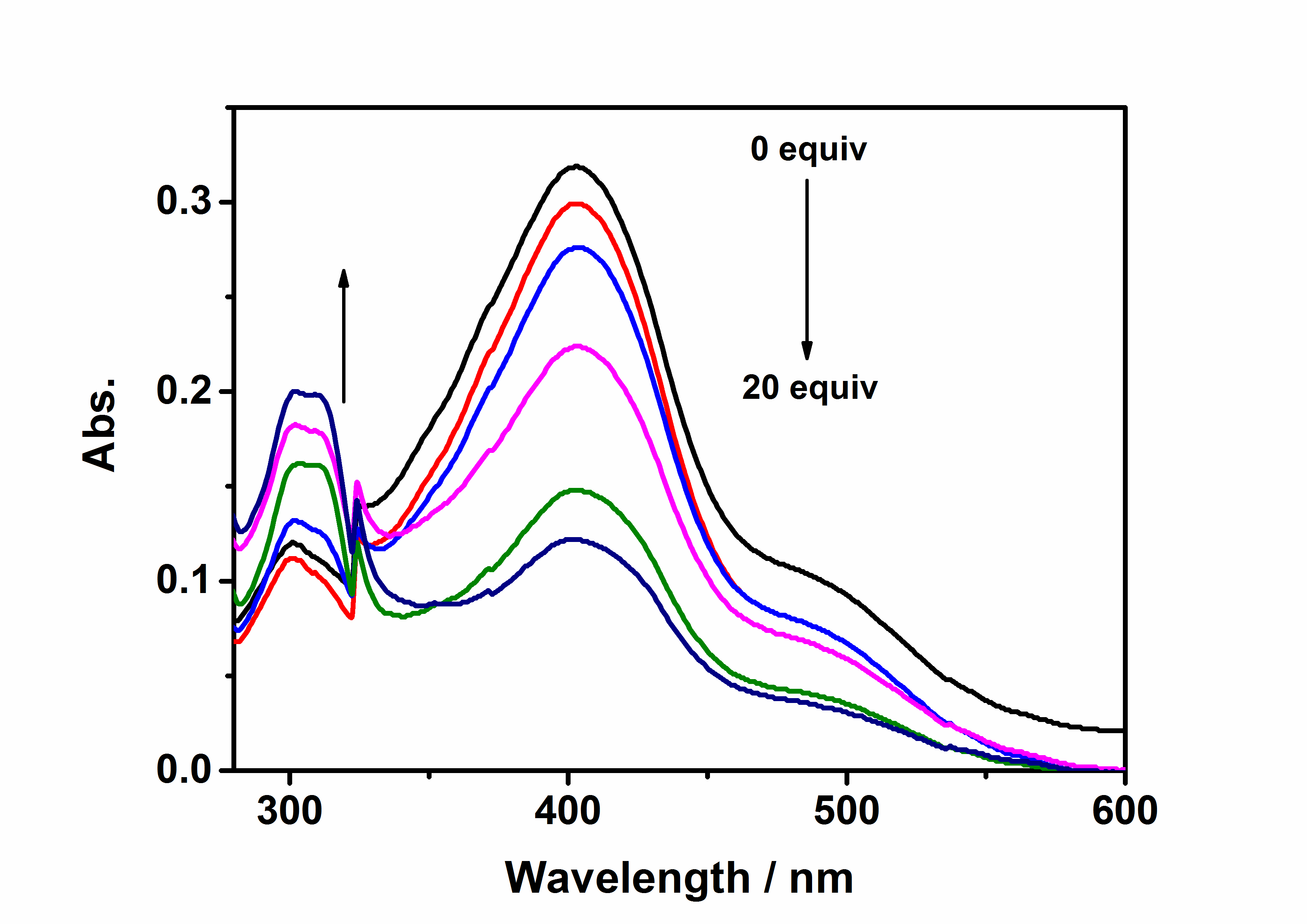


**Figure S1**. The absorption spectra of **NS-N_2_H_4_** (10 μM) in pH 7.4 PBS/DMSO (v/v = 2/1) in the absence or presence of N_2_H_4_ (20 equiv).


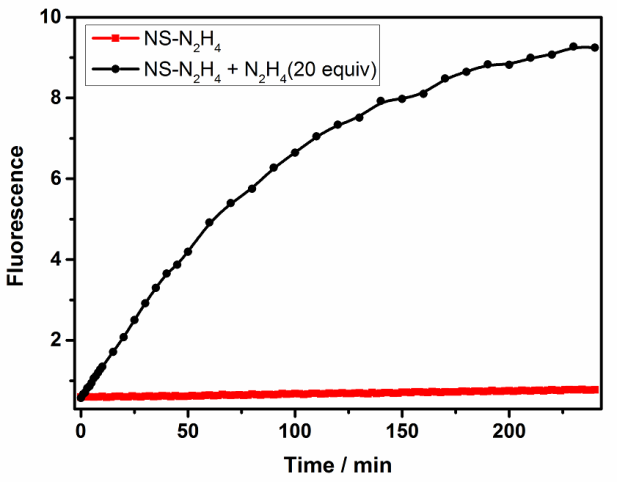


**Figure S2**. The stability of **NS-N_2_H_4_** probe under irradiation.


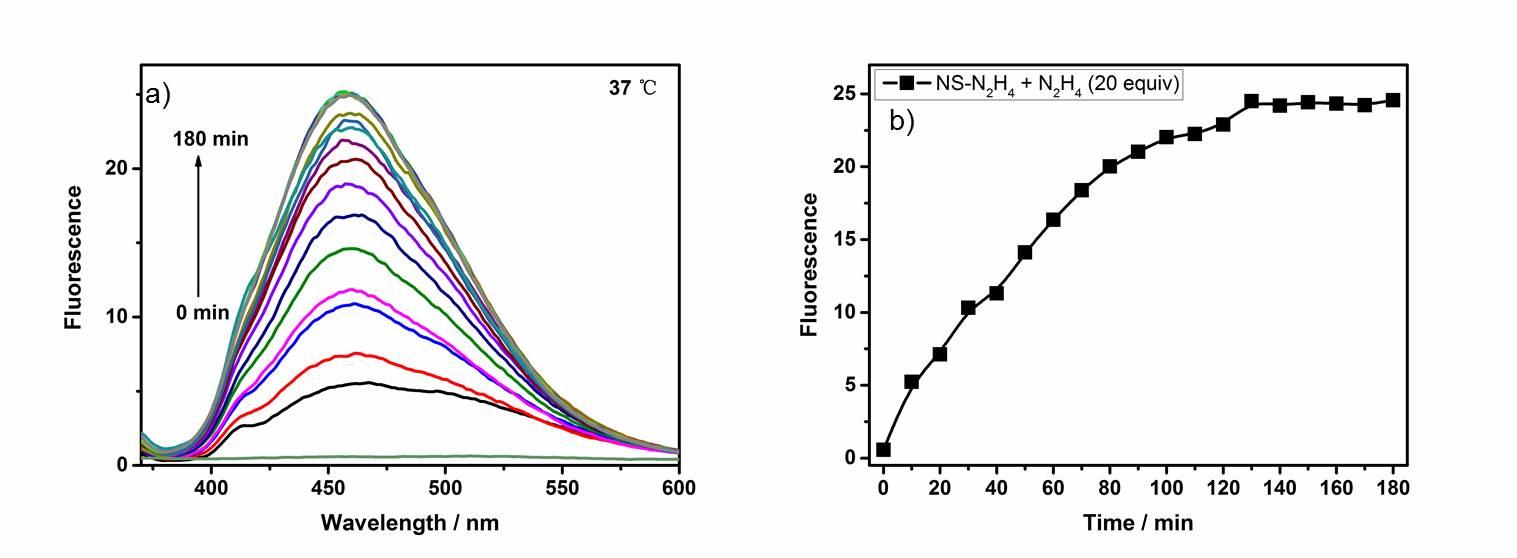


Figure S3. Fluorescence spectra of NS-N_2_H_4_ (10 μM) in pH 7.4 PBS/DMSO (v/v = 2/1) in the absence or presence of N_2_H_4_ at 37 °C.

**Figure S4**. HRMS (positive ion mode) spectrum of **NS-N_2_H_4_** (20 μM) after treatment with N_2_H_4_ (400 μM) in pH 7.4 PBS/DMSO (2: 1) for 60 min. The peak at m/z 187.0875 corresponds to **NS-N_2_H_4_**-adduct.


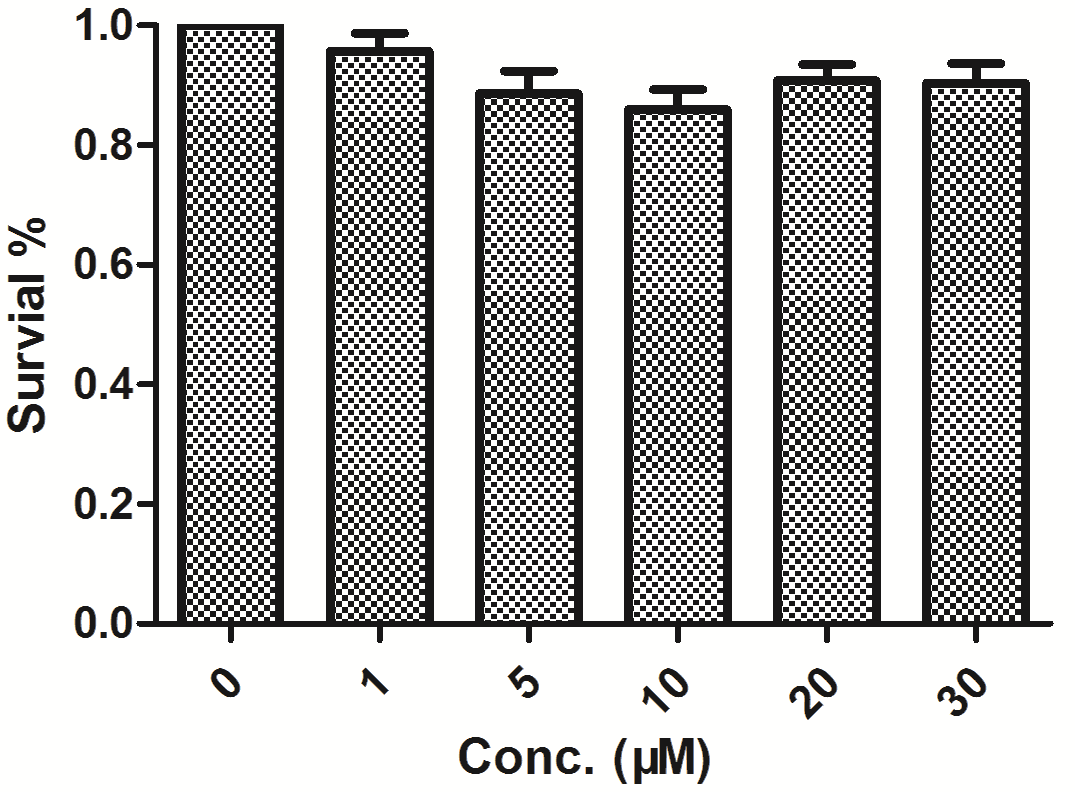


**Figure S5**. Cytotoxicity assays of **NS-N_2_H_4_** at different concentrations (0 μΜ; 1μΜ; 5 μΜ; 10 μΜ; 20 μΜ; 30 μΜ) for HeLa cells


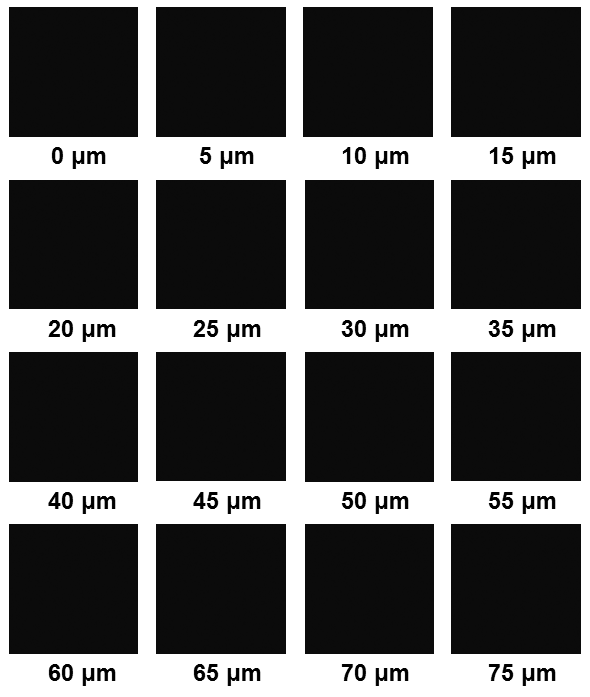


**Figure S6**. Two-photon fluorescence images of a fresh mouse liver slice incubated with **NS-N_2_H_4_** probe (20.0 μM) for 30 min in PBS buffer exhibit no fluorescence at the emission window of 0-75 nm. Excitation at 800 nm with fs pulse.

**Figure S7**. ^1^H NMR (DMSO*-d_6_*) spectrum of **NS-N_2_H_4_.**

**Figure S8**. ^13^C-NMR (DMSO*-d_6_*) spectrum of **NS-N_2_H_4_**.

**Figure S9**. HRMS spectrum of the probe **NS-N_2_H_4_**.
